# Correlation between the Macronutrient Content of Dental Calculus and the FFQ-Based Nutritional Intake of Obese and Normal-Weight Individuals

**DOI:** 10.1155/2021/5579208

**Published:** 2021-09-06

**Authors:** Ignatius Setiawan, Dian Lesmana, Dewi Marhaeni Diah Herawati, Irna Sufiawati, Sunardhi Widyaputra

**Affiliations:** ^1^Department of Public Health, Faculty of Dentistry, Maranatha Christian University, Bandung 40164, Indonesia; ^2^Department of Basic Medical Science, Faculty of Dentistry, Maranatha Christian University, Bandung 40164, Indonesia; ^3^Department of Public Health, Faculty of Medicine, Universitas Padjadjaran, Sumedang 45363, Indonesia; ^4^Department of Oral Medicine, Faculty of Dentistry, Universitas Padjadjaran, Sumedang 45363, Indonesia; ^5^Department of Oral Biology, Faculty of Dentistry, Universitas Padjadjaran, Sumedang 45363, Indonesia

## Abstract

The growing epidemic of chronic diseases afflicting both developed and developing countries is related to diet and lifestyle. The current dietary assessment still has many constraints, particularly related to the objectivity of data gathering. Dental calculus, which is usually considered as medical waste in dental treatment, turns out to be a provider of abundant oral information. The objective of this study is to obtain the correlation between the macronutrient content of dental calculus and nutritional intake based on FFQ. This research is an analytic observational study with a case-control study design. Samples consisting of 35 obese individuals and 21 normal-weight individuals were taken using purposive sampling. The nutritional intake data were obtained using FFQ. The macronutrient content of dental calculus was checked using a colorimetric assay. The comparison between obese individuals and normal-weight individuals was tested using the Mann–Whitney test and *T*-test. The correlation between the macronutrient content of dental calculus and nutritional intake based on FFQ was measured using Spearman's rank-order correlation. The results showed there was a correlation between the macronutrient content of dental calculus and macronutrient intake based on FFQ. However, strong correlation was found only between fat intake with the total lipid content of dental calculus with rs = 0.521 and between carbohydrate intake with the total carbohydrate content of dental calculus with rs = 0.519. It was concluded that carbohydrate, protein, and lipid intake can be assessed using dental calculus. Dental calculus can be an alternative source of noninvasive, inexpensive, and specific dietary biomarkers.

## 1. Introduction

The World Health Organization also estimates that globally, there are >1 billion overweight adults, 300 million of whom are obese [1]. Obesity and overweight increase the risk of several serious chronic diseases, such as type 2 diabetes, cardiovascular disease, hypertension and stroke, hypercholesterolemia, hypertriglyceridemia, arthritis, asthma, and certain forms of cancer [2]. Experts at the WHO and FAO found that the growing epidemic of chronic diseases afflicting both developed and developing countries is related to diet and lifestyle. Diet and nutrition are important factors in the promotion and maintenance of good health throughout the entire life course. Their role as determinants of chronic NCDs is well established, and they, therefore, occupy a prominent position in prevention activities [3].

Collecting dietary intake data is associated with many challenges, which are primarily related to the subjective nature of data collection tools such as Food Frequency Questionnaires (FFQs), multiple-day food records, and 24-hour dietary recalls [4]. Assessment of individuals' nutrition and energy intake patterns can be carried out by using the Food Frequency Questionnaire (FFQ). The FFQ is often designed to elicit information on specific aspects of the diet. In order to get data on the type and amount of nutritional intake, the appropriate FFQ to use is the semiquantitative FFQ. The semiquantitative FFQ is a qualitative FFQ with additional estimates of food portion sizes [5].

The current dietary assessment still has many constraints, particularly related to the objectivity of data gathering tools such as food frequency questionnaires (FFQs), multiple-day food records, and 24-hour dietary recalls [4, 5]. The most common constraint is that the respondents do not always remember all food that they have consumed or they do not know the specific content of the food (e.g., sandwich content), and it is difficult to determine an accurate portion size. In short, the respondents generally do not report all food intake [4].

The limitation of the questionnaire-based assessments is only evident when sufficiently valid and accurate food intake measurement is required [6]. This limitation can be overcome by using dietary biomarkers, which can assess food consumption objectively and which has a lower level of bias than the self-reported assessment. The need for dietary biomarkers is recognized by medical institutions as a knowledge gap that requires future research [4].

The potential of dietary biomarkers is needed to obtain an objective assessment of nutritional intake so as to improve the reliability of the study. The ultimate goal is to derive a statistical model that incorporates nutritional data that are based on biomarkers with those that are based on self-reported questionnaires [7]. Dietary biomarkers are not without limitations. High cost and invasive levels are important factors to consider; therefore, a noninvasive, inexpensive, and specific source of dietary biomarkers is very much needed [4]. Dietary biomarkers can be categorized into short term, medium term, and long term [4]. The dietary biomarkers can be obtained through biochemical measurements of the nutrient content of biological fluid and biological tissue, as well as the levels of excretion of nutrients or their metabolites [8]. Such biomarkers could be used to quantify intake and validate intake questionnaires, analyse physiological or pathological responses to certain food components or diets, identify persons with specific dietary deficiency, provide information on interindividual variations, or help to formulate personalized dietary recommendations to achieve optimal health for particular phenotypes, currently referred as “precision nutrition.” [9].

Dental calculus from fossils of ancient human teeth can be analysed to obtain data about the types of food as well as the cooking and processing technique [10, 11]. Another study has also shown evidence that ancient human beings consumed milk based on their dental calculus content [12]. Because of its location within the mouth, dental calculus offers a direct path to the material that has been inhaled or ingested [10].

Dental calculus is a complex, mineralized bacterial biofilm formed on the surfaces of teeth, principally from dental plaque but also with additional contributions from the saliva and gingival crevicular fluid. Biofilm formation begins when salivary protein is deposited as a thin film on the surface of the teeth, forming the acquired enamel pellicle (AEP). During human life, the AEP serves as a primary barrier and defensive layer between the calcium phosphate mineral of the enamel and bacterial and dietary acids [13]. Dental calculus is mainly composed of calcium phosphate mineral salts that are stored between and within the remnants of previously active microorganisms. The dental plaque covers the mineralized dental calculus. The level and location of dental calculus formations are specific for each population and are influenced by oral hygiene habits, access to professional care, diet, age, ethnic origin, time since the last dental cleaning, systemic disease, and prescription drug use [8].

Bacteria in dental plaque are related closely to the host. They use endogenous nutrients such as saliva, and glycoprotein proteins such as mucin, for their growth. The bacteria also produce a little acid and help to remove exogenous microorganisms [10]. Dental calculus is also an important source of microbiome information, which can provide accurate information about the evolution of microbiome, diet, and human health. Various diseases including obesity are found to be associated with changes in human microbiome [12].

Both supragingival and subgingival dental calculus are present in most adults worldwide. Dental calculus is formed by bacteria and calcium phosphate salts that combine to form the calculus. Subgingival calculus is useful for analysis as it accumulates and endures indefinitely if it is not mechanically removed [10].

There are two different categories of nutrients: the macronutrient and the micronutrient. The macronutrient which includes carbohydrate, protein, and lipid is a nutrient that is needed by the human body in large amount [14]. The macronutrient content in food can be trapped inside the dental calculus. The dental calculus, which is usually considered as a medical waste of dental treatment, turns out to be a provider of abundant oral information of long-term oral microbiotics, as well as food and environmental debris information [13].

The preliminary study was conducted to assess of carbohydrate, protein, and lipid content of dental calculus. There were three groups of dental calculus samples. The first group was treated with the Anthron method to assess the carbohydrate content. The second group was treated with the Soxhlet extraction method. The third group was treated with SDS-PAGE. Twenty samples of dental calculus were taken using the quota sampling method, each of which was taken from the medical waste from the patient who got dental calculus cleaning treatment at Maranatha Dental Hospital. The result showed that carbohydrates, proteins, and fats can be examined from dental calculus. Protein concentration was also measured in this preliminary study using a spectrophotometer (Bio Drop), then protein was examined with the western blot technique using SDS-PAGE electrophoresis, and after that, the proteins were transferred from the gel onto the PVDF membrane for antibody staining and detection. Bands were found in the staining results [15].

Based on the abovementioned description, the authors intend to conduct a research to assess the content of carbohydrates, proteins, and lipids of dental calculus, so that it can be a source of long-term dietary pattern information. This study aims to obtain the correlation between macronutrient intake based on the FFQ and the content of macronutrients of dental calculus in obese and normal-weight individuals.

## 2. Materials and Methods

This study is an analytic observational study with a case-control study design. The data in this study were obtained from filling out the Food Frequency Questionnaire and the assessment of the macronutrient content of dental calculus. Then, data from obese individuals were compared to data from normal-weight individuals. The data were collected From Jan 2019–Jan 2020. We used the STROBE checklist of items that should be included in reports of case-control studies for designing and conducting studies.

The population in this study was the people of Bandung and its surroundings. There were also patients who attended Maranatha Dental Hospital. For this study, they were divided into two groups. The first group is the case group, a group of individuals with obesity. The second group is the control group, a group of normal-weight individuals. The participants were selected using the quota sampling method. The sample size, meaning the number of participants, was obtained with the sample size formula without the correction factor by Fleiss. According to a study conducted by Nikmah and Dany in 2017, the prevalence of obese individuals having high leptin levels (percent of exposed with outcome) is 69% and the prevalence of high leptin level in normal-weight individuals (percent of unexposed with outcome) is 32% [16]. Based on these data and by using a significance level of 95%, a power value (1-beta, % chance of detecting) of 80%, ratio of control to cases of 0.6, and odds ratio of 4.8, it could be calculated and found that the minimum participant number for the exposed (case) group is 35 individuals and for the unexposed (control) group is 21 individuals [17].

The inclusion criteria for the participant in the case group were patients aged 18–40 years, indicated for dental calculus cleaning, a body mass index score of ≥25, and a body lipid percentage of ≥25% for men and ≥33% for women. The participant inclusion criteria in the control group were patients aged 18–40 years, indicated for dental calculus cleaning, a body mass index score of 18.5–22.99, and a body lipid percentage of 8–15% for men and 13–23% for women. The exclusion criteria for the participants in both the case and control groups were as follows: first, patients who had conditions that made it impossible to participate in the study, for instance, mental disorders and diseases with a high risk of infection; second, patients who had conditions that interfered with the assessment of body mass index scores, for instance, pregnancy, and a weight loss program in the past 1 year. In this study, to avoid bias, body weight, height, and body lipid percentage of all participants were measured using the same tool.

Dental calculus in this study was only taken from individuals who were medically indicated for dental calculus cleaning; therefore, the dental calculus was medical waste from dental calculus cleaning performed by professionals at Maranatha Dental Hospital. After collection, dental calculus was then rinsed with 70% alcohol and then stored in a sterile tube.

Nutritional intake was assessed using the semiquantitative FFQ. The FFQ is a self-reporting method of measuring food intake through filling out a questionnaire. Each study participant reports how often each type of food and drink is consumed during a certain period of time [5, 14]. In the semiquantitative FFQ, the amount of nutrients present in each type of food consumed can be calculated based on the results of a questionnaire. The list of foods used in the FFQ is obtained from preliminary research using a food record.

The assessment procedure was as follows: First, the participants were required to fill in the list provided in the questionnaire regarding the frequency of their intake. There were 5 categories of intake frequency: daily (D), weekly (W), monthly (M), yearly (Y), and rarely/never (N). The participants selected the most appropriate category for the frequency of consumption of each type of food and wrote the number of times each food was consumed in the boxes provided. Second, the participants were also required to mark on the list provided in the questionnaire regarding the intake portion. There were 3 categories of intake portion which indicate the quantity of food usually consumed: small (S), medium (M), and large (L). The food book photo from the Indonesian Ministry of Health was used as the standard measure of food portion [18]. Third, to measure daily nutrient intake, all categories of frequency were converted into a daily basis, which meant once a day = 1. The daily frequency was multiplied by the selected portion (in grams) to get the weight in grams consumed each day. The assessment of nutritional intake was conducted by the same person to avoid bias.

This study was conducted according to the guidelines laid down in the Declaration of Helsinki, and all procedures involving human subjects/patients were approved by the Health Research Ethics Committee, Faculty of Medicine, Universitas Padjadjaran, Bandung, Indonesia (No. 119/UN6.KEP/EC/2019). Written informed consent was obtained from all subjects/patients.

### 2.1. Total Carbohydrate Measurement

Total carbohydrate was measured using the phenol-sulfuric acid method. In this method, the concentrated sulfuric acid broke down any polysaccharides, oligosaccharides, and disaccharides into monosaccharides. Pentoses (5-carbon compounds) were then dehydrated to form furfural and hexoses (6-carbon compounds) to form hydroxymethyl furfural. These compounds then reacted with phenol to produce a golden yellow colour [19]. The absorption was then measured using a spectrophotometer at a wavelength of 490 nm. The glucose was used to create the standard curve. The colour for this reaction is stable for several hours, and the accuracy of the method was within ±2% under proper conditions.

First, glucose standards were made for colorimetric detection. An amount of 0, 2, 4, 6, 8, and 10 *µ*L of the 2 mg/mL standard solution was added directly to each of 96 wells in the plate to generate standards of 0 (blank), 4, 8, 12, 16, and 20 *µ*g/well. Water was added to each well to bring the volume to 30 *µ*L.

An amount of 0.5 mg of the dental calculus sample from each participant was prepared. The dental calculus samples were then homogenized in a 20 *µ*L assay buffer. After that, the samples were centrifuged at 13,000x g for 5 minutes to remove any insoluble material. Next, the sample was added with aquabidest to obtain a final volume of 30 *µ*L.

As much as 30 *µ*L of the standard solution and sample solution was each added to separate well. Then, 150 *µ*L of concentrated H_2_SO_4_ was added to each well, and then, the solution was homogenized using a horizontal shaker. After that, the plate was incubated for 15 minutes at 90°C. The plates were ensured to be closed and not exposed to light during incubation. Then, 30 *µ*L of developer was added to each well, and the solution was homogenized again using a horizontal shaker for 6 minutes at room temperature. The colour change indicates the presence of carbohydrates, and then, the absorbance was measured at a wavelength of 490 nm using a spectrophotometer. The measurement was repeated 3 times for the sample solution.

### 2.2. Total Lipid Measurement

The total lipid content of the dental calculus sample was measured using the sulfo-phospho-vanillin (SPV) method. The SPV reaction was performed in two steps, the initial reaction of the lipids with concentrated sulfuric acid at high temperature followed by the second reaction of the derived products with vanillin in the presence of phosphoric acid [20].

Consensus understanding is that a positive SPV reaction requires the presence of double bonds or free hydroxyl groups within the lipid analytes. The chemical reactions are complex and are thought to involve the formation of relatively stable carbonium ion (or carbocation) chromogens (alkenyl cations) in the initial reaction followed by generation of pink chromophore upon the addition of vanillin to the reaction. The colour change was measured at a wavelength of 540 nm using a spectrophotometer.

To make the standard solution, a purified lipid was prepared at a concentration of 100 g/dL. The solution was then diluted to 2500, 1250, 625, 313, 156, 78, 39, and 0 (mg/dL). The sample was prepared using the Folch method. An amount of 0.5 mg dental calculus sample was dissolved in 200 *µ*L of methanol and then incubated for 1 hour at 1600 rpm at room temperature. Afterwards, 400 *µ*L of chloroform was added and then incubated again for 2 hours at 1600 rpm at room temperature. Next, the supernatant was taken and then added with 200 *µ*L of 0.9% NaCl and centrifuged at 3000 rpm for 5 minutes. The bottom layer was taken and measured.

As much as 15 *µ*L of the standard solution and sample solution were each added to separate well. Then, 150 *µ*L of concentrated H_2_SO_4_ was added to each well, and then, the solution was homogenized using a horizontal shaker. After that, the solution was incubated for 10 minutes at 90°C. The plates were ensured to be sealed and not exposed to light during incubation. Next, 100 *µ*L of the solution that had been incubated was taken and added into another well and measured at a wavelength of 540 nm to determine the blank. Afterwards, 100 *µ*L of vanillin reagent was added and the solution was homogenized again. The solution was then incubated again at 37°C for 15 minutes. Then, the absorbance was measured at a wavelength of 540 nm using a spectrophotometer. The measurement for the sample solution was carried out 3 times.

### 2.3. Total Protein Measurement

The protein content was measured using the Bradford method. The most conspicuous advantages of the Bradford method are the use of a single reactive, the rapidity of the reaction (just 5 min.), a high level of stability of the protein-dye complex, a high level of reproducibility, and the occurrence of minimal interferences. The Bradford method is based on the absorbance shift observed in an acidic solution of dye Coomassie® Brilliant Blue G-250. When added to a solution of protein, the dye binds to the protein resulting in a colour change from reddish brown to blue [21]. Dyes come in three forms: cationic (red), neutral (green), and anionic (blue). Under acidic conditions, the dye is predominantly double-protonated red in its cationic form (470 nm). However, when the dye binds to the protein, it turns blue (595 nm). The blue solution on the microplate is measured using a spectrophotometer or microplate reader.

The extinction coefficient of a dye-albumin complex solution is constant over a 10-fold concentration range; thus, Beer's law may be applied for accurate quantification of protein by selecting an appropriate ratio of dye volume to sample concentration.

To make the standard solution, a stock solution of commercial bovine serum albumin (BSA) was prepared at the concentration of 2000 *μ*g/mL. Then, 6.67 *µ*L of the BSA solution was diluted using 993.33 *µ*L of aquabidest. After that, a series of dilutions from 200 to 0 was made. As much as 20 *µ*L of the standard solution was added to the well plate. Dental calculus sample preparation was the same as the sample preparation for total carbohydrate measurement. An amount of 0.5 mg of the dental calculus sample from each participant was prepared. The dental calculus samples were then homogenized in a 20 *µ*L assay buffer. After that, the samples were centrifuged at 13,000x g for 5 minutes to remove any insoluble material. Next, each sample was added with aquabidest to obtain a final volume of 30 *µ*L.

An amount of 20 *µ*l of the sample solution was added to the well plate. Afterwards, 200 *µ*L of Quick Start Dye Reagent was also added to each well plate. The solution turned blue. The solution was then incubated at room temperature for 5 minutes, after which the absorbance was measured at 595 nm.

## 3. Results

The range of body mass index (BMI) of obese individuals in this study was 26.1–44.7 kg/m^2^, whereas the average BMI is 34,2 kg/m^2^. For normal-weight individuals, the range of BMI was 17.1–23.5 kg/m^2^ whereas the average BMI was 19.8 kg/m^2^. The percentage of body fat of the participants in the case group was 42.7%, while the average percentage of body fat of the participants in the control group was 19.9% (see Table 1).

The assessment of dietary intake using the FFQ showed the results in Table 2. The average value of carbohydrate intake of obese individuals was 288.6 g, while for normal-weight individuals, it was 226.3 g. Those data showed that obese individuals had larger carbohydrate intake than normal-weight individuals although both obese and normal-weight individuals in this study exceeded the average limit of normal carbohydrate intake for adults, which is 130 g/day [22]. The average value of protein intake of obese individuals was 88.4 g, while for normal-weight individuals, it was 78.4 g. The average value of lipid intake of obese individuals was 77.3 g, while for normal-weight individuals, it was 75.5 g. The average value of total energy intake of obese individuals was 2161.8 kcal, while for normal-weight individuals, it was 1873.1 kcal.

The assessment of the macronutrient content of dental calculus showed the following results. The average value of the total carbohydrate content of dental calculus of obese individuals was 0.30089 mg/ml, while for normal-weight individuals, it was 0.29219 mg/ml. The average value of the total protein content of dental calculus of obese individuals was 0.93583 mg/ml, while for normal-weight individuals, it was 0.85214 mg/ml. The average value of the total lipid content of dental calculus of obese individuals was 1.06840 mg/ml, while for normal-weight individuals, it was 1.08148 mg/ml. Those data showed that obese individuals had higher total carbohydrate and total protein content of dental calculus than normal-weight individuals, which was in line with the result from the FFQ. Both assessments showed a higher value for obese individuals than for normal-weight individuals. Meanwhile, the value of total lipid of dental calculus of obese individuals was lower than it was for normal-weight individuals. The complete data are shown Table 3.

The comparison of carbohydrate intake and total energy intake between obese and normal-weight individuals was tested using the *T*-Test. Mean scores (SD) were used for comparison of carbohydrates, whereas median value was used for comparison of total energy. The comparison of protein intake and fat intake between obese and normal-weight individuals was tested using the Mann–Whitney test. The range value was used for the test. Based on the *p* value, only the carbohydrate intake showed a significant difference between obese and normal-weight individuals (*p* value = 0.016). Meanwhile, the comparison of protein, fat, and total energy intake between obese and normal-weight individuals did not show a significant difference. The data are shown in Table 4.

The comparison of the macronutrient content of dental calculus between obese individuals and normal-weight individuals was tested using the nonparametric Mann–Whitney test. The *p* value of total carbohydrate, total protein, and total lipid was found to be >0,05 (see Table 5). It means that there were not any significant differences of macronutrient content between obese individuals and normal-weight individuals.

The correlation between the macronutrient content of dental calculus and nutritional intake based on the FFQ was measured using Spearman's rank-order correlation. Spearman's correlation coefficient, (*ρ*, also signified by rs) measures the strength and direction of correlation between two ranked variables. The Spearman correlation coefficient, rs, can take values from 1 to −1. An rs of 1 indicates a perfect correlation of ranks, an rs of zero indicates no correlation between ranks, and an rs of −1 indicates a perfect negative correlation of ranks. The closer the rs value is to zero, the weaker the correlation between the ranks. The data showed that there was a correlation between each macronutrient content of dental calculus and macronutrient intake based on the FFQ. However, strong correlation was only found between fat intake and the total lipid content of dental calculus with rs = 0.521 and between carbohydrate intake and the total carbohydrate content of dental calculus with rs = 0.519. Based on the *p* value, only carbohydrate and lipid had a significant correlation between the intake value based on the FFQ and the value of its content in dental calculus (see Table 6).

## 4. Discussion

FFQ, food diaries, and 24 h recall methods represent the most commonly used dietary assessment tools in human studies on nutrition and health, but food intake biomarkers are assumed to provide a more objective reflection of intake. Unfortunately, very few of these biomarkers are sufficiently validated [23]. The Food Frequency Questionnaire is a retrospective method, based on the memory of the respondent and most often concerns a longer period of time, e.g., one month or one year, and, therefore, represents a habitual diet [5]. Short-term dietary intake indicates the dietary intake of the previous few hours/days, medium-term dietary intake indicates the dietary intake of the previous several weeks, and long-term dietary intake indicates the dietary intake of the previous several months/years [4]. The biochemical analysis of a reference metabolite that indicates the bioavailability of a nutrient is an objective nutritional status assessment method, which allows less methodological error and detects deficiency states more precisely than dietary assessment [9].

Dental calculus, a mineralized form of dental plaque, serves as a long-term reservoir of dietary biomolecules and microfossils [24]. Nearly ubiquitous in archaeological populations and sourced directly from the oral cavity, dental calculus presents a unique opportunity to access primary evidence of ancient diets at an individual level [24]. Dietary reconstructions based on plant microfossils, such as starch grains and phytoliths, have also been useful in increasing our understanding of past human populations [25]. The development of dental calculus is a dynamic process that starts with a nonmineralized biofilm which eventually calcifies. The nonmineralized dental biofilm entraps particles from the oral cavity, including large amounts of oral bacteria, human proteins, viruses, and food remnants, and preserves their DNA [26].

Dental plaque is a dense mass of bacteria, also known as the biofilm, which is tightly adherent to the tooth surface. Bacterial attachment to the tooth is mediated by receptors in the thin-layer salivary coating of the tooth surface, termed the acquired pellicle. The pellicle and plaque matrix are composed of host-derived and bacterial products. Bacteria in the dental plaque have a close relationship with the host, they use endogenous nutrients such as saliva and glycoprotein proteins such as mucin, for their growth, from which there is little acid production, and their presence helps remove exogenous microorganisms (colonization resistance) [27].

Dental plaque is formed in the presence of sucrose or glucose and fructose. Its relation to cariogenicity was evaluated, and the results suggested that high level of cariogenicity occurs when dental plaque is formed in the presence of sucrose [28]. Dental calculus is indeed a stable, long-term reservoir of proteins as previously reported, but further systematic studies are needed to identify mechanisms associated with protein entrapment and survival in dental calculus [29].

Based on the abovementioned explanation, dental calculus can be an alternative source of long-term nutritional intake information. The mineralized matrix of dental calculus is of high physical hardness and durability, preserving organic microscopic debris and biomolecules. Frequently found on skeletal material, calculus has been described as “one of the richest known sources of ancient biomolecules in the archaeological record,” preserving molecular evidence of oral bacteria and the human host, as well as consumed foodstuffs, all of which can be directly tied to the individual [13, 29, 30].

Not all data from the macronutrient content of dental calculus were found inline with the result from the FFQ; therefore, further study to determine the specific content of nutrient as a dietary biomarker in dental calculus is needed. Some limitations were also considered in this study:

- The participant number for the exposed (case) group and unexposed (control) was different. However, the calculation of the number of samples was conducted in accordance with statistical rules.
- The sample size was moderate and originated from the same source; hence, our results cannot be generalized. Future studies required a larger and various sample size.
- The dental calculus samples were medical waste from dental calculus cleaning performed by professionals at Maranatha Dental Hospital; therefore, the dental calculus collector was not calibrated. However, the dental calculus cleaning is a standard procedure in dentistry, and the professionals in Maranatha Dental Hospital conducted their job according to applicable standard medical procedures.
- The kappa value for assessment of nutritional intake was not implemented; however, the assessment was conducted by the same person to avoid bias.

## 5. Conclusions

This study has succeeded in examining the macronutrient content of dental calculus in modern humans. The conclusion of this study is carbohydrate, protein, and lipid can be assessed from dental calculus. There is a correlation between the macronutrient content of dental calculus and macronutrient intake based on the FFQ. Strong correlation was only found between fat intake and the total lipid content of dental calculus and between carbohydrate intake and the total carbohydrate content of dental calculus. Dental calculus can be an alternative source of noninvasive, inexpensive, and specific dietary biomarker.

## Figures and Tables

**Table 1:** Descriptive statistics of body fat percentage and BMI in both obese and normal-weight individuals.

		Statistical measure			*p* value
		Mean (SD)	Median	Range	
Body fat percentage (%)	Obese	42.7 (8.8)	43.5	27.7–58.7	0.148
	Normal weight	19.9 (7.4)	20.4	5.0–30.1	0.211

Body mass index (kg/m^2^)	Obese	34.2 (4.4)	33.4	26.1–44.7	0.11
	Normal weight	19.8 (1.7)	19.8	17.1–23.5	0.825

**Table 2:** Descriptive statistics of macronutrient intake based on the FFQ in both obese and normal-weight individuals.

FFQ		Statistical measure			*p* value
		Mean (SD)	Median	Range	
Carbohydrate intake (g)	Obese	288.6 (121.3)	310.6	96.3–589.9	0.128
	Normal weight	226.3 (65.7)	245.3	126.3–344.8	0.131

Protein intake (g)	Obese	88.4 (35.7)	82.8	42–215	0.003
	Normal weight	78.4 (32.7)	70.4	23.4–143.3	0.617

Fat intake (g)	Obese	77.3 (36.6)	71.6	23.1–157.6	0.022
	Normal weight	75.5 (39.2)	65.5	12.9–175.8	0.666

Total energy intake (g)	Obese	2161.8 (788.1)	2165.7	820.2–4250	0.490
	Normal weight	1873.1 (632.5)	1852.3	736.4–3169	0.693

**Table 3:** Descriptive statistics of the macronutrient content of dental calculus of both obese and normal-weight individuals.

Content of dental calculus		Statistical measure			*p* value
		Mean (SD)	Median	Range	
Total carbohydrate (mg/ml)	Obese	0.30089 (0.0307)	0.294	0.260–0.407	0,002
	Normal weight	0.29219 (0.0484)	0.281	0.228–0.411	0,220
	All participants	0.29763 (0.0381)	0.292	0.228–0.411	

Total protein (mg/ml)	Obese	0.93583 (0.2785)	0.864	0.175–1.767	0,010
	Normal weight	0.85214 (0.2292)	0.808	0.441–1.312	0,869
	All participants	0.90445 (0.2622)	0.863	0.175–1.767	

Total lipid (mg/ml)	Obese	1.06840 (0.0507)	1.061	0.982–1.230	<0,001
	Normal weight	1.08148 (0.0411)	1.072	1.016–1.184	0,005
	All participants	1.07330 (0.0474)	1.070	0.982–1.230	

**Table 4:** Comparison of nutritional intake based on the FFQ between obese and normal-weight individuals.

Intake based on the FFQ	Group		*p* value
	Obese (*n* = 35)	Normal weight (*n* = 21)	
Carbohydrate (g)	288.6 (121.2)	226.3 (65.7)	0.016
Protein (g)	82.8 (42.0–215.0)	70.4 (23.4–143.3)	0.402
Fat (g)	71.6 (23.1–157.6)	65.5 (12.9–175.8)	0.993
Total energy (kcal)	2161.8 (788.1)	1873.1 (632.5)	0.160

**Table 5:** Comparison of the macronutrient content of dental calculus between obese and normal-weight individuals.

Content of dental calculus		Group		*p* value
		Obese (*n* = 35)	Normal weight (*n* = 21)	
Total carbohydrate (mg/ml)	Median	0.294	0.281	0.257
	Range	(0.260–0.407)	(0.228–0.411)	

Total protein (mg/ml)	Median	0.864	0.808	0.198
	Range	(0.175–1.767)	(0.441–1.312)	

Total lipid (mg/ml)	Median	1.061	1.072	0.121
	Range	(0.982–1.230)	(1.016–1.184)	

**Table 6:** Correlation of nutrition intake based on the FFQ and macronutrient content in dental calculus between obese and normal-weight individuals.

Intake based on the FFQ		Macronutrient content of dental calculus					
		Total carbohydrate (mg/ml)		Total protein (mg/ml)		Total lipid (mg/ml)	
		Obese	Normal weight	Obese	Normal weight	Obese	Normal weight
Carbohydrate intake (g)	Correlation coefficient	0.157	0.519	—		—	
	Sig. (2-tailed)	0.367	0.016	—		—	
Protein intake (g)	Correlation coefficient	—		0.258	0.004	—	
	Sig. (2-tailed)	—		0.135	0.987	—	
Fat intake (g)	Correlation coefficient	—		—		0.246	0.521
	Sig. (2-tailed)	—		—		0.154	0.015

## Data Availability

The.xlsx data used to support the findings of this study are available from the corresponding author upon request.
